# Raman Investigation on Silicon Nitride Chips after Soldering onto Copper Substrates

**DOI:** 10.3390/mi15080990

**Published:** 2024-07-31

**Authors:** Claudia Mezzalira, Fosca Conti, Danilo Pedron, Raffaella Signorini

**Affiliations:** 1 Department of Chemical Science, University of Padova, Via Marzolo 1, I-35131 Padova, Italy; claudia.mezzalira9@gmail.com (C.M.); danilo.pedron@unipd.it (D.P.); 2 Consorzio INSTM, Via G. Giusti 9, I-50121 Firenze, Italy

**Keywords:** sintering, bonding, cracking, interconnections, silicon nitride, reliability, assembly, stress and strain, Raman

## Abstract

The unique electrical properties of silicon nitride have increased the applications in microelectronics, especially in the manufacture of integrated circuits. Silicon nitride is mainly used as a passivation barrier against water and sodium ion diffusion and as an electrical insulator between polysilicon layers in capacitors. The interface with different materials, like semiconductors and metals, through soldering may induce residual strains in the final assembly. Therefore, the dentification and quantification of strain becomes strategically important in optimizing processes to enhance the performance, duration, and reliability of devices. This work analyzes the thermomechanical local strain of semiconductor materials used to realize optoelectronic components. The strain induced in the β-Si_3_N_4_ chips by the soldering process performed with AuSn pre-formed on copper substrates is investigated by Raman spectroscopy in a temperature range of −50 to 180 °C. The variation in the position of the E_1g_ Raman peak allows the calculation of the local stress present in the active layer, from which the strain induced during the assembly process can be determined. The main reason for the strain is attributed to the differences in thermal expansion coefficients among the various materials involved, particularly between the chip, the interconnection material, and the substrate. Micro-Raman spectroscopy allows for the assessment of how different materials and assembly processes impact the strain, enabling more informed decisions to optimize the overall device structure.

## 1. Introduction

The interest in ceramic materials is constantly growing due to the widespread uses in most industries in the world. Characteristic advantages of ceramics are strength in compression, strong chemical stability against erosion, and high resistance to a very large temperature range [1]. 

Silicon nitride (Si_3_N_4_) is a covalently bonded ceramic compound characterized by superior strengthening behaviour, enhanced tribological performance, and anti-corrosion activities [2]. The ceramic form is a polycrystalline material with hexagonal crystal structure. The crystal system is generally divided into α and β crystal directions [3]. However, among them, β-Si_3_N_4_ is the thermodynamically more stable and therefore more common phase [4]. The β-structure has a phenacite assembly morphology and consists of a trigonal arrangement of silicon atoms tetrahedrally bonded to four nitrogen atoms to form corner-sharing SiN_4_ tetrahedra [5]. Si_3_N_4_ crystallizes in the hexagonal C^2^_6h_(P6_3_/m) space group [6,7]. The microstructure consists of elongated crystals that interlock into micro-rods, with the consequence of having very low density (3.2 g∙cm^−3^ at 25 °C) and good flexural strength (850 MPa) [2]. Si_3_N_4_ has a pale grey colour and is characterized by chemical resistance and high thermal stability, with a melting point of 1900 °C for the powder. Because of the high thermal shock resistance, Si_3_N_4_ has a high fracture toughness of almost 7 MPa∙m^1/2^ [2]. It is a rather expensive material. But, in relation to its performance in applications, the cost–benefit ratio is excellent, especially where it can outperform the normally utilized materials, with a long life and very reliable low maintenance operation. Thanks to these properties, the material is widely exploited for different life science applications [8], ranging from medicine [9,10] to integrated photonics [11,12,13]. Most uses are as high-temperature and high-load engineering materials for gas turbines, diesel engines, abrasives, i.e., where the toughest conditions are demanded [14]. However, its use is also important in the production of computers, electronic product manufacturing, and photonic and optoelectronic components [15,16].

Low propagation loss, high power handling, and CMOS compatibility are typical reasons why silicon nitride is used in photonic-integrated circuit applications [17]. Some of the most promising photonic technologies are quantum applications, LIDAR, high-performance light sources, spectroscopy, gas sensing, and in the communication domain [18,19]. Recently, Ma et al. have introduced a method to obtain a complete photonic bandgap in a Si_3_N_4_ photonic crystal slab [20]. 

Independently from technological use, in every configuration it is necessary to have silicon nitride interconnected to other materials. The integration of these components on a common substrate addresses issues associated with parasitic capacitances and inductances caused by separate physical connections, thereby enhancing circuit responsiveness [21]. In microelectronic packaging [22], mainstream processes are bonding, soldering, sintering or conductive adhesive bonding on substrates. The main requirement for all time remains the reliability of the contacts. To guarantee effective interconnected technologies, stress phenomena in the assemblies must be reduced. Internal stresses have the potential to deform the microstructure of the device, sometimes leading to its destruction. For this reason, the evaluation of the mechanical properties of materials within these devices is deemed crucial for both performance and reliability considerations [23].

Raman spectroscopy is a valuable tool to investigate the perturbations produced in the materials after interconnection, induced principally by thermal mismatch of the different materials [24,25,26,27,28]. It has the advantages of being a fast, noncontact, and nondestructive technique with a micrometer spatial resolution [29]. The principle behind the correlation of the Raman bands and the local stress is found, considering that a mechanical or thermal stress affects the frequencies of the optical phonons, through a displacement of the atoms involved in their production from their starting equilibrium positions. If the crystal is perturbated, after an interconnecting procedure, the Raman peaks undergo a shift, which can be correlated with the stress spreading on the material [30,31]. 

Other methods used to characterize residual stresses in thin films present several drawbacks: the substrate curvature method is basically based on the detection of the change in the curvature of the substrate before and after the film deposition [31]. It provides a stress value averaged over the film area, but owing to its approximate algorithms of stress distribution, it is suitable neither for the residual stress measurement of thick films nor for films with asymmetric stress distributions and is not useful when information must be obtained on local stress. X-ray diffraction examines the residual stresses in different depths of the film structure owing to the penetration capability of X-rays but does not satisfy the requirement of mechanical measurement on microelectronic devices due to its inherent limitations in spatial resolution and accuracy [32]. Cross-sectional transmission electron microscopy (XTEM) and convergent beam electron diffraction (CBED) [33] can provide information on stress on a very-high-resolution scale (nm), but destructive sample preparation is required, causing stress relaxation in certain directions, and the interpretation of XTEM images needs extensive modeling. Because of these considerations, the Raman technique may be considered the most suitable technique for the purpose. 

Group theory predicts 11 Raman active modes for β-Si_3_N_4_ [6,7]. If the crystal is perturbated, like after an interconnecting procedure, then the Raman peaks undergo a shift, which can be correlated to the stress spreading on the material.

In this paper, micro-Raman spectroscopy is used to investigate the local stress induced from the assembly process of silicon nitride chips. Thanks to its micrometrical resolution, the Raman technique has been exploited to collect the signal in different positions of the sample to achieve a complete evaluation of the stress distribution. This paper analyses the strain induced in β-Si_3_N_4_ by the soldering process performed with preformed AuSn [34,35]. From the recorded spectra, the variation in the position of the E_1g_ Raman peak allows the calculation of the local stress present in the active layer, from which the strain induced during the assembly process can be determined. 

## 2. Materials and Methods

### 2.1. Substrates, Interconnections, and Chips

Copper substrates with dimensions of 5 × 5 × 1 mm^3^ were prepared by rubbing the surfaces on grain-abrasive discs, ensuring thorough cleaning without causing scratches. Subsequently, the substrates underwent ultrasonic baths in 4% HCl and isopropanol to further improve cleanliness.

**Figure 1:**
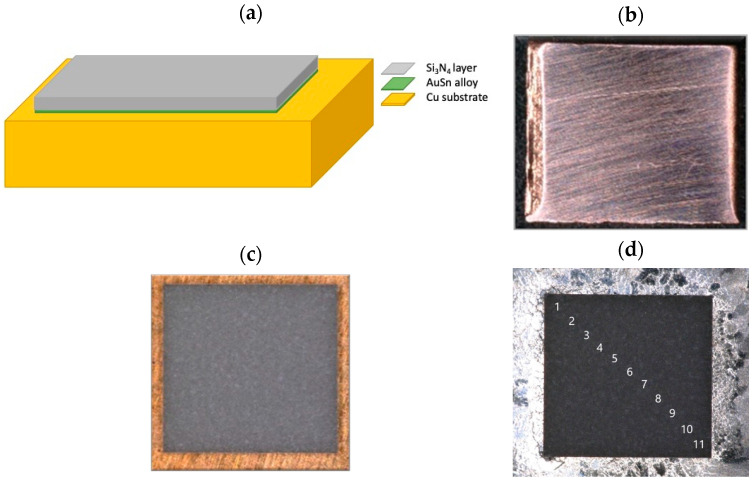
Schemes of assembly (**a**) and photos of the top view (**b**–**d**): Cu substrate before assembling, (**b**) Si_3_N_4_ chip after assembling with pressure (**c**), assembly with identification of the positions for Raman measurements along the diagonal of the sample (**d**). Each position (1–11), named with a number, represents the area where a Raman signal was acquired.

To assemble the devices (see Figure 1), interconnections were established using a soldering process that involved a thin layer (25 μm thick) of the Au80Sn20 eutectic alloy [36,37]. Silicon nitride chips measuring 1500 × 1500 × 120 μm^3^, with (001) orientation and featuring gold metallization on the bottom face, were adopted.

**Figure 2:**
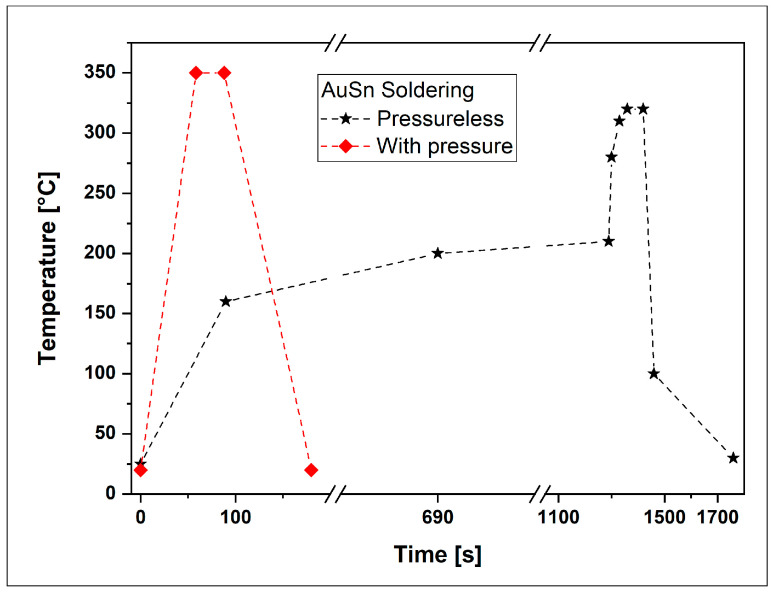
Temperature profile used for the soldering process with an AuSn alloy as interconnection material, without pressure (**black stars**) and under 5N pressure (**red diamond**).

The soldering process was executed in two conditions according to the pressure parameter. The samples prepared without pressure underwent an oven treatment with a controlled atmosphere and a temperature profile, as outlined in Figure 2, adhering to the process conditions detailed in Table 1. The soldering process employed for samples assembled with 5 N pressure utilized a Fineplacer (FineTech AP2.4 145/FP 145 Pico ma, AP2.4 145/FP 145 Pico ma, Finetech GmbH, Berlin, Germany). 

This equipment positions the chip and exerts a specified pressure perpendicular to the substrate surface while simultaneously controlling the system’s environment through heating. The chosen pressure of 5 N represents the maximum tolerable pressure to prevent the silicon nitride chip from breaking during the assembly process. The temperature profile for soldering under pressure is also illustrated in Figure 2 and Table 2 outlines the specific process conditions.

### 2.2. Samples

Table 3 lists all prepared samples. The two unmounted samples refer to silicon nitride chips not connected to the substrate, while the six Si_3_N_4_ chips were mounted on Cu substrates using a soldering process with either no pressure (3 samples) or 5 N pressure (3 samples). The chip and substrate dimensions and the applied pressure are also reported.

### 2.3. Methods

The optical characterization of the samples involved recording Raman spectra across numerous positions on the chip surface, spanning a wide temperature range. 

#### 2.3.1. Micro-Raman Set-Up

The Raman instrument consisted of a micro-Raman spectroscope operating in a back-scattering configuration. An argon ion laser with a monochromatic wavelength of 514.5 nm (Spectra Physics Stabilite 2017, output power 1 W, Spectra-Physics^®^ Milpitas, California) served as excitation source. The laser radiation was regulated by a pass-band interference filter, which selectively allowed only 514.5 nm radiation. The incident light’s polarization was managed by a half-wave plate. A system of mirrors and lenses collimated and directed the radiation to a set of Optical Density (OD) filters. A beam splitter divided the radiation, sending 50% to an optical microscope (Olympus BX 40, Tokyo, Japan). The microscope was equipped with interchangeable lenses (4×, 10×, 20×, 50×, 100×) and with a sample holder table, which was able to move in three directions (x, y, z) to adjust the position of the sample. A camera was integrated with the microscope to observe and precisely select the sample area interacting with the laser [38]. The typicalI spot diameter of the laser beam at the focus was 3 μm. The backscattered radiation passed through a notch filter to remove the Rayleigh (elastic) component of the diffused radiation and through a lens to focus the beam on the slit of the spectrograph. Then, the radiation traversed an 1800 grid and was converted into an electronic signal by a Charge Coupled Device (CCD). Finally, the collected signal underwent processing, amplification, and digital conversion before being transferred to the PC memory. For micro-Raman measurements at specific temperatures, a cryostat with a controlled temperature chamber (Linkam THMS600, Tadworth, UK) was employed. A nitrogen environment was created, preventing issues arising from water particles in the chamber.

#### 2.3.2. Micro-Raman Acquisition Parameters

To record the Raman signal of the silicon nitride samples, an acquisition time of 25 s was required, with averaging through 5 repeated measurements (25 × 5). Due to the high acquisition time required to obtain spectra with a good signal-to-noise ratio for silicon nitride samples, 11 points along the diagonal of the chip were measured, as shown in Figure 1. The main reason for the point selection was to study the evolution of stress phenomena from the corner to the center of the chip. Each point was recorded using the 20× lens. The spectrograph slit remained constant at 40 μm throughout the analysis of all samples, and the applied OD filter was 03, corresponding to an input power of ~15 mW on the sample.

#### 2.3.3. Sample Temperature Controlling

Raman spectra were obtained at various temperatures. Initially, the first measurement was conducted at room temperature for all the samples. Subsequently, the Raman set up was equipped with a temperature controller to facilitate spectrum recording at different temperatures, spanning a range from −50 °C to 180 °C.

#### 2.3.4. Calibration of Raman Spectra

For the accurate calibration of the spectrograph, a standard commercial silicon sample Raman signal was initially employed. Additionally, all spectra of the reported samples were normalized in relation to the plasma line signal. This normalization was performed to ensure that the position of the band of interest remained unaffected by instrumental variations.

#### 2.3.5. Determination of Stress from Raman Spectra

Piezo spectroscopy was employed for assessing residual stresses in ceramic materials, such as silicon nitride. The methodological approach was based on the relationship between the shift in the Raman peak position and the residual stress values of the samples when subjected to small strains [7,39]. When an unstressed material, initially with a Raman signal centered at $\omega_{0}$, undergoes a stress field, the peak shifts to a different frequency, $\omega_{j}$. This shift is described in Equation (1) by Taylor’s series expansion of $\omega_{0}$:

(1) $$\omega_{j}=\omega_{0}+{\left(\frac{{\delta \omega }_{j}}{{\delta \sigma }_{ij}}\right)}_{ij}\sigma_{ij}+\frac{1}{2}{\left(\frac{{\delta^{2}\omega }_{j}}{{\delta \sigma }_{ij}{\delta \sigma }_{kl}}\right)}_{ijkl}\sigma_{ij}\sigma_{kl}+\ldots$$

where $\sigma_{ij}$ represents the stress field in the crystallographic lattice’s reference system. As the second derivative term was much smaller than the first ones, it was neglected in the expansion [40]. To approximate the stress dependence from the investigated peak, the matrix of the first term in Taylor’s series needs to be determined, as indicated in Equation (2):

(2) $$\left(\frac{{\delta \omega }_{j}}{{\delta \sigma }_{ij}}\right)=\frac{\omega_{j}-\omega_{0}}{\sigma_{ij}}$$

These elements are commonly referred to as the first-order piezo spectroscopic coefficients, $\Pi_{ij}$, and represent the constant of proportionality between the Raman band shift $\Delta \omega_{j}$ and the residual stress $\sigma_{ij}$, as expressed in Equation (3):

(3) $$\Pi_{ij}=\frac{\Delta \omega_{j}}{\sigma_{ij}}$$

$\Pi_{ij}$ was determined with a calibration technique, inferring a calibration sample at a known (external) stress field, which can have uniaxial, biaxial, or hydrostatic characteristics, and then monitoring the spectral shift of selected Raman bands. Then, the slope of the experimentally generated calibration plot of the Raman band position against the magnitude of applied stress corresponds to the value of $\Pi_{ij}$ [41]. Finally, stress values were calculated using the following Equation (4):

(4) $$\sigma_{ij}=\frac{\Delta \omega_{j}}{\Pi_{ij}}$$

**Figure 3:**
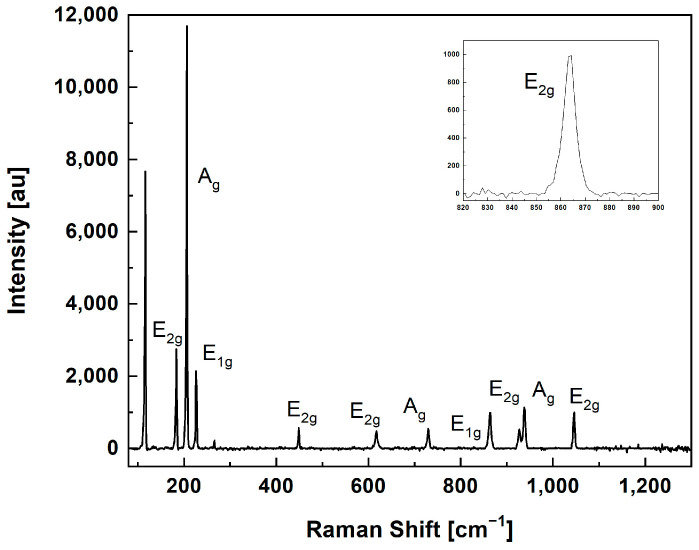
Experimental silicon nitride Raman spectrum.

For the equi-biaxial stress of β silicon nitride, the coefficient assumed a value of $\Pi_{ij}=-4.2\pm 0.3\;{cm}^{-1}{GPa}^{-1}$ [31]. This value was used to assess induced stress using the band at a frequency of 866 cm^−1^ (reported in Figure 3), corresponding to the E_1g_ mode, as it exhibited the highest variation in position.

The stress model adopted in this work considered a uniform biaxial stress along the [001] and [010] axes (xy plane). Stress was quantified starting from the backscattering micro-Raman measurement along the [001] direction (z-axis) of the (001) silicon or silicon nitride surface; referring to the selection rules [7,24,42] and considering that the laser radiation was polarized along the (1–10) plane, the stress measurement was related to the variation in the peak associated with the LO phonon (z-polarized). The z direction (or [001]) corresponded to the direction of incidence of the laser beam, which was perpendicular to the surface of the chip. In conclusion, Equation (4) was used to correlate the peak shift to the stress values. The evaluation of induced stress in chips through the assembly process involved considering the unmounted sample as a reference (providing the value of $\omega_{0}$) and measuring the Raman shift position of the assemblies.

## 3. Results

### 3.1. Raman Spectrum of Si_3_N_4_

The Raman spectrum of the unmounted Si_3_N_4_ samples is reported in Figure 3. From the Raman spectrum of silicon nitride, it is possible to observe the plasma lines at 116.3 and 265.5 cm^−1^ and the silicon nitride peaks. 

The peak frequencies are reported in Table 4, together with the Raman mode assignment. From the comparison with the experimental and theoretical modes reported in the literature [6,7,43], it is possible to find a good agreement of the frequencies.

### 3.2. Behavior of Raman Signals along the Diagonal of the Samples

**Figure 4:**
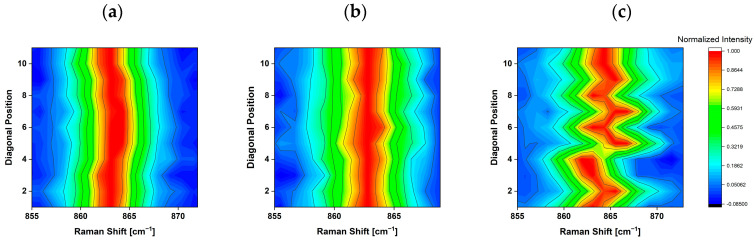
E_1g_ peak position of the Raman spectra at room temperature of diagonal points of N_Unmounted (**a**), N_A_AuSn_p (**b**), and N_A_AuSn (**c**).

Raman measurements on silicon nitride samples were carried out on eleven diagonal positions of the chip. The main reason for the selection was the analysis of the results obtained with the silicon samples [44] because it was evident that the study of the diagonal represents the most interesting way to evaluate the stress distribution on the chip. In Figure 4, the E_1g_ peak position of the Raman spectra measured at room temperature along the diagonal of the silicon nitride samples is reported. 

The unmounted silicon nitride sample does not show an appreciable change in the peak position along the diagonal corresponding to the highest variation in Raman shift of 0.4 cm^−1^. N_A_AuSn_p shows a slight shift of the peak positions, with the highest variation in the Raman shift of 0.8 cm^−1^, slightly higher than the unmounted sample. The N_A_AuSn sample was assembled without pressure and shows a Raman shift distribution along the diagonal almost symmetrical with respect to the central position, characterized by the highest variation in Raman shift of about 2 cm^−1^.

### 3.3. Effect of Temperature on Si_3_N_4_ Samples

**Figure 5:**
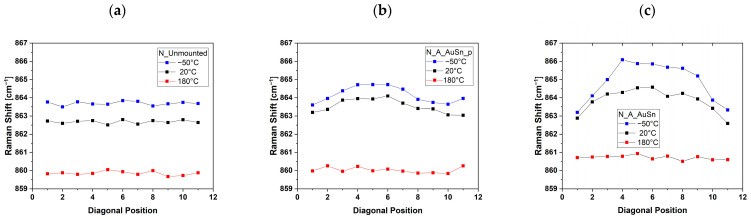
E_1g_ peak position on the diagonal of N_Unmounted (**a**), N_A_AuSn_p (**b**), and N_A_AuSn (**c**) at room temperature (**black dots**), at −50 °C (**blue dots**) and 180 °C (**red dots**).

The Raman shift of the E_1g_ peak position along the diagonal at different temperatures was determined by fitting the Raman spectra in the range centered at 860 cm^−1^ with a Lorenzian function. The values are reported in Figure 5 for the N-Unmounted (Figure 5a), N_A_AuSn_p (Figure 5b), and N_A_AuSn (Figure 5c) samples. The N-Unmounted sample has a Raman signal that oscillates around 863.6 cm^−1^ at room temperature, and the value increases up to 863.7 cm^−1^ at −50 °C, while it drops to 862.8 cm^−1^ as the temperature increases to 180 °C. No variations in the Raman shift were observed as a function of the position along the diagonal of the sample. For the mounted samples, however, the Raman shift values at the extremes of the diagonal were similar to those of the unmounted samples, while they showed an increase along the diagonal until reaching the maximum value at the central position. 

Figure 5b,c show an almost symmetric distribution of the Raman shift with respect to position 6 of the diagonal.

**Figure 6:**
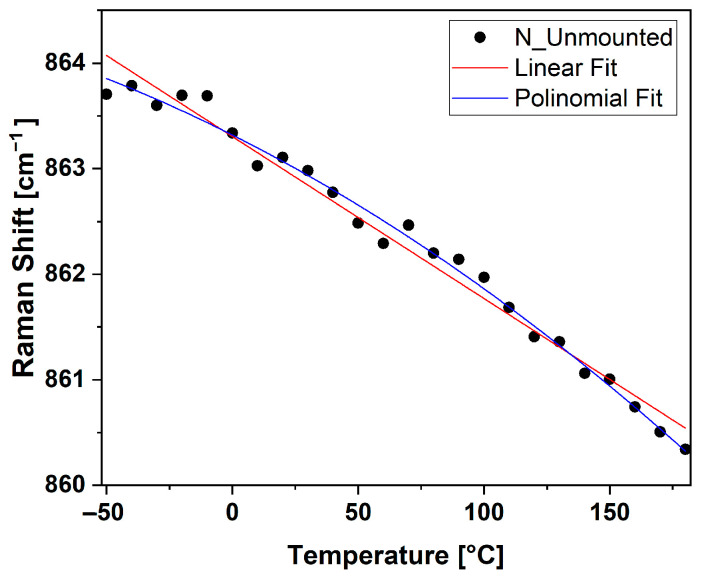
Raman shift collected in position 6 of N_Unmounted sample at temperatures ranging from −50 °C to 180 °C. Lines represent the curve fitting using a linear (**red**) or polynomial (**blue**) regression.

The Raman signal of the E_1g_ mode was measured at position 6 on the diagonal of the N-Unmounted sample at different temperatures ranging from −50 to 180 °C with ΔT = 10 °C to verify that the trend was continuous in the temperature interval considered. The data are reported in Figure 6, together with the linear and a second-order polynomial regression, as reported in articles [45,46]. The polynomial expression is A + Bx + Cx^2^, where A is the Raman shift when the temperature is extrapolated to 0 K, and B and C are thefirst- and second-order temperature coefficients, respectively.

Considering the values of the coefficient of determination, R^2^, the polynomial fit was better in describing the trend of the data. The linear fit was obtained using an intercept of 863.30 ±0.05 cm^−1^, a slope of −0.0154 ±0.0005, and an R^2^ = 0.978. The polynomial fit was obtained using an intercept of 863.32 ±0.03 cm^−1^, a slope of −0.0120 ±0.0003, and an R^2^ = 0.989.

## 4. Discussion

The stress values of the samples were calculated using Equation (4), where $\omega_{j}$ was chosen as the Raman shift value of the peak corresponding to the E_1g_ mode. For the N-Unmounted samples, $\omega_{0}$ was chosen as the average Raman shift of all the peaks of the diagonal, while for the assembled ones, $\omega_{0}$ was chosen as the mean value of the N-Unmounted. 

The unmounted samples presented stress values randomly distributed on the diagonal of the chip, within an interval ranging less than 100 MPa. From the calculation of the uncertainties of the stress values, it emerged that the measured stress values were affected by an experimental error of the same order of magnitude of the calculated errors. This point agrees with previous expectations since the unmounted samples were used as zero-stress reference materials and stress values around zero were expected. These values represented the starting point for calculating the stress of the other samples.

The data of the calculated stress of the unmounted sample, together with the corresponding Raman shift values, determined at all temperatures are reported in Table 5, where the maximum, the minimum, and the average value of Raman shift and stress are displayed for temperatures of 50 °C, 20 °C, and 180 °C, with their respective uncertainties. The highest variations in Raman shift and stress at the three temperatures are also reported. The stress values of the unmounted samples were around zero and more precisely between −47 and 43 MPa at 180 °C and even lower at room temperature (−28 to 40 MPa) and −50 °C (−36 to 46 MPa). 

**Figure 7:**
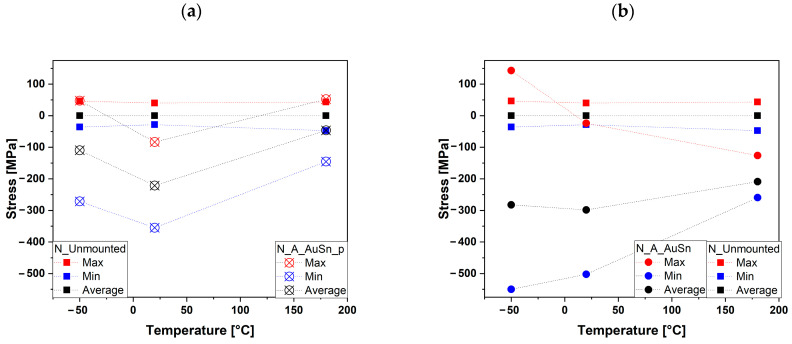
The strain developed on N-Unmounted sample (squares) and N_A_AuSn_p (X with circles) samples (**a**) and N-Unmounted sample (squares) and N_A_AuSn (circles) samples (**b**) as a function of temperature. The red and blue symbols refer to the minimum and maximum found values, respectively. The black symbols refer to the averaged strain developed.

The highest stress values were obtained for the sample assembled without pressure. The corresponding data are reported in Table 6 and described in Figure 7a; at low temperature, the stress value shift from the tensile stress of 142 MPa to the compressive stress of −550 MPa, while at room temperature and at high temperature the stress is compressive and ranges between −24 and −502 MPa and −126 and −259 MPa, respectively.

The values of the sample assembled with pressure are reported in Table 7 and described in Figure 7b; at −50 and 180 °C, the stress shifts from a small tensile (47 and 52 MPa) to a larger compressive stress (−271 and −145 MPa respectively), while at 20 °C it ranges from −83 to −355 MPa. The assembled samples show mainly a compressive stress phenomenon at each considered temperature.

The effect of the temperature changes on phononic frequencies was attributed to the different populations of vibration levels at each temperature. This effect is not further explored in the present paper because it is not related to the purpose of this study. For more information, the reader may refer to the article of Lucazeau et al. [47]. From the results obtained in this work, it was clear that the stress affected the phonon frequencies at different temperatures. Raman shift profiles for the silicon nitride sample positions calculated at different temperatures showed the best fitted applying a second-degree polynomial equation. It demonstrated that the effect of the temperature had different consequences on the samples, which depends on their stress values.

The temperature effects were also studied for the silicon nitride unmounted sample, and the profiles were best fitted using a second-degree polynomial equation.

The strain variation in the Si_3_N_4_ samples shows a distinct dependency on temperature, with much lower strain values at 180 °C and the highest strain variations observed at −50 °C. This behavior can be attributed to the thermal history and the properties of the materials (reported in Table 8) involved in the sample assembly process.

During the soldering process, which typically occurs between 200 °C and 350 °C, both the chip and the substrate are characterized by a less rigid form, with closed cells in a less stiff configuration due to the high temperature. The solder, in its liquid form, connects the components. As the assembly cools down, each material contracts according to its distinctive and specific thermal expansion coefficient. The different thermodynamics and kinetics of the contraction process lead to strain development within the assembly. The key factors contributing to the observed strain variations are (i) thermal expansion coefficients, (ii) elastic and plastic deformation, and (iii) temperature dependence of material properties. Different materials in the assembly, such as the Si_3_N_4_ layer, the substrate, and the solder, have distinct thermal expansion coefficients, reported in Table 8. The literature shows that copper has a high thermal expansion coefficient (15–20 ppm/°C) compared to silicon nitride (around 5–7 ppm/°C), while Au80Sn20 alloy and gold possess high thermal expansion coefficients (16 and 14.3 ppm/°C, respectively). As the temperature decreases from the soldering range to room temperature or lower, each material shrinks at a different rate, creating internal stresses.

Moreover, during cooling, a temperature regime is possible where the solder remains soft and can plastically deform to accommodate the shrinking components. However, once the solder solidifies and the assembly reaches a lower temperature, mechanical rigidity is established. At this point, any further temperature decrease induces elastic strain in the materials, reflected in the Si_3_N_4_ layer. The mechanical properties of materials, such as Young’s modulus and yield strength, can vary with temperature. At higher temperatures (e.g., 180 °C), materials may exhibit more ductile behavior, allowing for some strain relaxation. Conversely, at lower temperatures (e.g., −50 °C), materials tend to be more brittle, leading to higher strain accumulation.

The lower strain variations at 180 °C can be explained by the combination of more ductile behavior of materials and the possibility of plastic deformation in the solder during cooling. In contrast, at −50 °C, the materials are more brittle, and the different thermal contraction results in higher elastic strain in the Si_3_N_4_ layer. These considerations are crucial for optimizing the thermal management and mechanical reliability of Si_3_N_4_-based devices. The results at different temperatures clearly show that the strain variation in the Si_3_N_4_ layer is characterized by much lower values when measured at 180 °C. On the contrary, the highest strain variations were recorded at −50 °C. A possible explanation is suggested by considering the sample assembly processes: for soldered samples, the temperature is mainly between 220 °C and 250 °C. This means that liquid solder connects an expanded chip to an expanded substrate at these temperatures. As the cooling ramp begins, all materials shrink according to their thermal expansion coefficient, which differs within the chip components, between the chip and the interconnect, and between the interconnect and the substrate. Even if the existence of a temperature range for a solder with a still-soft interconnect that can plastically adapt to the shrinking sizes of the sample components is assumed, mechanical rigidity is achieved, and elastic strain is built into the chip.

**Figure 8:**
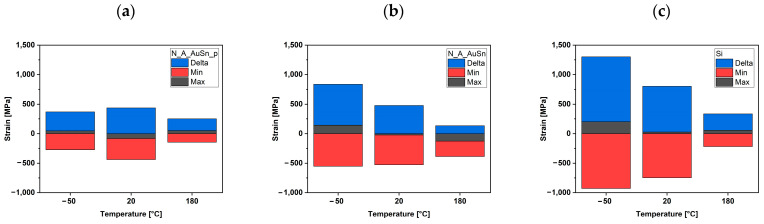
Maximum (**black**) and minimum (**red**) strains developed on N_A_AuSn_p (**a**) and N_A_AuSn (**b**) samples and differences—Delta (**blue**) between them as a function of the temperature. Comparison with strain developed in Si sample (**c**), adapted from Ref. [44].

The comparison between the Si_3_N_4_ samples and the Si sample [44], illustrated in Figure 8, highlights several key insights into the effects of material on the strain developed within the assemblies. The following considerations can be summarized. The Si_3_N_4_ sample, assembled without pressure, exhibits significant differences in stress values compared to the Si assembly. At low and room temperatures, the difference in maximum stress values between the two assemblies is approximately 400 MPa, reducing to around 90 MPa at 180 °C. The difference is greater for the samples assembled with pressure: 800 MPa at −50 °C, 500 MPa at 20 °C, and 80 MPa at 180 °C. The strong reduction in the stress difference at 180 °C suggests that the thermal expansion behavior and the mechanical properties of the materials are temperature-dependent. At higher temperatures, the materials may undergo more significant relaxation and plastic deformation, leading to a more uniform stress distribution. The choice of material and the experimental parameters used during the assembly process (e.g., soldering temperature and cooling rate) are crucial in determining the strains developed within the assembly. The interplay between these factors influences the thermal and mechanical stability of the final device.

## 5. Conclusions

Silicon nitride, initially recognized as a structural ceramic material, enables the production of high-quality components for extremely demanding applications. Its commercial interest began to increase in the 1950s, leading to its development in various refractory applications. Its self-reinforced microstructure, exceptionally high strength, toughness, and excellent thermal properties—such as minimal expansion and contraction due to temperature changes and the ability to withstand thermal shock—make it an attractive structural component for various industries. These include automotive, bearing, aviation and aerospace, optics and electronics, and in recent years, biomedical and biosensing applications, thanks to its biocompatibility.

In the field of electronics, the need for increasingly advanced performance necessitates high-quality control of assemblies, which translates into the control of stress developed within the assemblies. 

This study suggests practical applications in the design and optimization of optoelectronic devices. 

The use of micro-Raman spectroscopy as a tool for process control offers significant advantages. It allows for precise detection, localization, and quantification of stress and strain phenomena within the materials. It is a valid method for identifying crack formation and propagation in interconnect materials and joints, enabling the optimization of assembly processes. By monitoring the stress distribution in real time, adjustments can be made to the assembly parameters to minimize strain and enhance the reliability of the devices.

In summary, this work underscores the importance of carefully selecting materials and optimizing assembly parameters to manage stresses and strains in semiconductor devices. The ability to control these factors is critical for the performance and operating life of any microelectronics device. Micro-Raman spectroscopy proves to be a valuable tool in this optimization process, providing detailed insights into stress distribution and enabling more effective process control. The high sensitivity of the Raman method allows for the investigation of failure modes in electronic systems.

## Figures and Tables

**Table 1:** Pressureless soldering process with an AuSn alloy as interconnection material. On/off refers to the switching of the gas cooling system.

Duration mm:ss	Temperature [°C]	Ramp Rate [°C/s]	Cool	N_2_	Formic Acid
00:30	25	50	off	on	off
01:00	160	135	off	off	on
10:00	200	4	off	off	on
10:00	210	1	off	off	on
00:10	280	420	off	off	on
00:30	310	60	off	off	on
00:30	320	20	off	off	on
01:00	320	0	off	off	on
00:40	100	−330	on	on	off
05:00	30	−14	on	on	off

**Table 2:** Working parameters for the soldering process under pressure with an AuSn alloy as interconnection material. On/off refers to the switching of the gas cooling system.

Duration mm:ss	Temperature [°C]	Ramp Rate [°C/s]	Cool	N_2_
00:00	25	0	off	off
01:00	325	5	off	on
00:30	325	0	off	on
01:00	25	−3	on	off

**Table 3:** Samples and substrates with main characteristics.

Sample Name	Chip Dimensions [mm^3^]	Number of Investigated Samples	Cu Substrate Dimensions [mm^3^]	Pressure [N]
N_unmounted	1.5 × 1.5 × 0.12	2	-	0
N_A_AuSn_p	1.5 × 1.5 × 0.12	3	5 × 5 × 1	5
N_A_AuSn	1.5 × 1.5 × 0.12	3	5 × 5 × 1	0

**Table 4:** Raman mode frequencies of unmounted β-Si_3_N_4_.

Experimental Peak Frequency [cm^−1^]	FWHM [cm^−1^]	Raman Mode Assignment β-Si_3_N_4_	Literature Experimental Peak Frequency [cm^−1^] [6]	Literature Calculated Peak Frequency [cm^−1^] [7]
		*A_g_*	145	Not available
183.5	2.7	*E*_2*g*_	185	183
206.2	2.0	*A_g_*	208	201
226.2	2.7	*E*_1*g*_	230	228
		*A_g_*	Not available	444
448.9	2.1	*E*_2*g*_	452	457
617.1	5.1	*E*_2*g*_	620	603
729.5	4.1	*A* *_g_*	733	715
863.1	5.8	*E*_1*g*_	866	836
925.9	5.8	*E*_2*g*_	930	897
936.7	5.1	*A* *_g_*	940	908
1044.9	4.3	*E*_2*g*_	1048	1012

**Table 5:** Raman shift and stress values obtained for N_Unmounted sample at −50, 20, and 180 °C.

Temperature [°C]		Maximum Value	Minimum Value	Average Value	Maximum Variation
−50	Raman shift [cm^−1^]	863.8 ± 0.1	863.5 ± 0.1	863.7 ± 0.1	0.2
	Stress [MPa]	46 ± 24	−36 ± 24	0	82
20	Raman shift [cm^−1^]	862.8 ± 0.1	862.5 ± 0.1	862.7 ± 0.1	0.3
	Stress [MPa]	40 ± 24	−28 ± 24	0	68
180	Raman shift [cm^−1^]	860.0 ± 0.1	859.7 ± 0.1	859.8 ± 0.1	0.3
	Stress [MPa]	43 ± 24	−47 ± 24	0	90

**Table 6:** Raman shift and stress values obtained for N_A_AuSn sample at −50, 20, and 180 °C.

Temperature [°C]		Maximum Value	Minimum Value	Average Value	Maximum Variation
−50	Raman shift [cm^−1^]	866.1 ± 0.1	863.2 ± 0.1	864.9 ± 0.1	2.9
	Stress [MPa]	142 ± 26	−550 ± 45	−282 ± 25	692
20	Raman shift [cm^−1^]	864.6 ± 0.1	862.6 ± 0.1	863.9 ± 0.1	2
	Stress [MPa]	−24 ± 24	−502 ± 43	−298 ± 16	478
180	Raman shift [cm^−1^]	860.9 ± 0.1	860.5 ± 0.1	860.7 ± 0.1	0.5
	Stress [MPa]	−126 ± 25	−259 ± 30	−208 ± 24	133

**Table 7:** Raman shift and stress values obtained for N_A_AuSn_p sample at −50, 20, and 180 °C.

Temperature [°C]		Maximum Value	Minimum Value	Average Value	Maximum Variation
−50	Raman shift [cm^−1^]	864.7 ± 0.1	863.6 ± 0.1	864.2 ± 0.1	1.1
	Stress [MPa]	47 ± 24	−271 ± 30	−109 ± 31	318
20	Raman shift [cm^−1^]	864.1 ± 0.1	863.0 ± 0.1	863.5 ± 0.1	1.1
	Stress [MPa]	−83 ± 24	−355 ± 34	−221 ± 28	272
180	Raman shift [cm^−1^]	860.3 ± 0.1	859.8 ± 0.1	860.0 ± 0.1	0.8
	Stress [MPa]	52 ± 24	−145 ± 26	−46 ± 18	192

**Table 8:** Properties of the materials of the assembly components.

Material	Thermal Expansion Coefficient [ppm/°C]	Elastic Modulus [GPa]	References
Si_3_N_4_	5–7	120–240	[48,49]
Au	14.3	61	[50]
Cu	15–20	131	[35]
Al_2_O_3_	8.1	330	[36]
Au80Sn20 Alloy	16	68	[51]

## Data Availability

Data are contained within the article.
